# Supplementary material for: Engineering and characterization of gymnosperm sapwood toward enabling the design of water filtration devices
Source: Nat Commun. 2021 Mar 25;12:1871. doi: 10.1038/s41467-021-22055-w (PMC7994624; doi:10.1038/s41467-021-22055-w)
Supplement: Supplementary file 1 — Supplementary Information [file 41467_2021_22055_MOESM1_ESM.pdf]

Supplementary Information

Engineering and characterization of gymnosperm sapwood toward enabling the design of water filtration devices by Krithika Ramchander et al.

Contents

Supplementary Figures
Supplementary Fig. 1: Mechanism of ethanol-based dry preservation
Supplementary Fig. 2: Mechanism of self-fouling and its mitigation
Supplementary Fig. 3: Fouling behavior of xylem filters
Supplementary Fig. 4: Field validation of xylem filters
Supplementary Fig. 5: Xylem filter device prototypes
Supplementary Fig. 6: Cost comparison of xylem filters with commercial filters belonging to four major brands (denoted by different colors) in India shows the potential to offer unprecedentedly low cartridge replacement cost. Product names are included.

Supplementary Notes
Supplementary Note 1: Criteria for practically useful filters
Supplementary Note 2: Simulation of percolation effects in xylem filters
Supplementary Note 3: Ethanol treatment of xylem filters
Supplementary Note 4: Alternate hypotheses for drop in permeance of xylem filters in the absence of contaminants
Supplementary Note 5: Fouling model for xylem filters
Supplementary Note 6: Design of Granular Activated Carbon (GAC) column
Supplementary Note 7: Cost of manufacturing xylem filters
Supplementary Note 8: Resource requirement for fabricating xylem filters and filtration devices
Supplementary Note 9: Behavior change interventions for HWT adoption
Supplementary Note 10: Improvement in rejection ability due to fouling
Supplementary Note 11: Design guide for selecting tree species for making xylem filters
Supplementary Note 12: HWT for emergency use
Supplementary Note 13: Potential avenues for engagement of micro-enterprises and local communities in xylem filter manufacture and distribution

Supplementary Tables
Supplementary Table 1: Microbiological performance data for xylem filters
Supplementary Table 2: Water quality parameters for field tests

Supplementary Data
Supplementary Data 1: Geographic availability, structural and degradation characteristics, and pricing of gymnosperms (provided separately as Excel file)

Supplementary References

Supplementary Figures

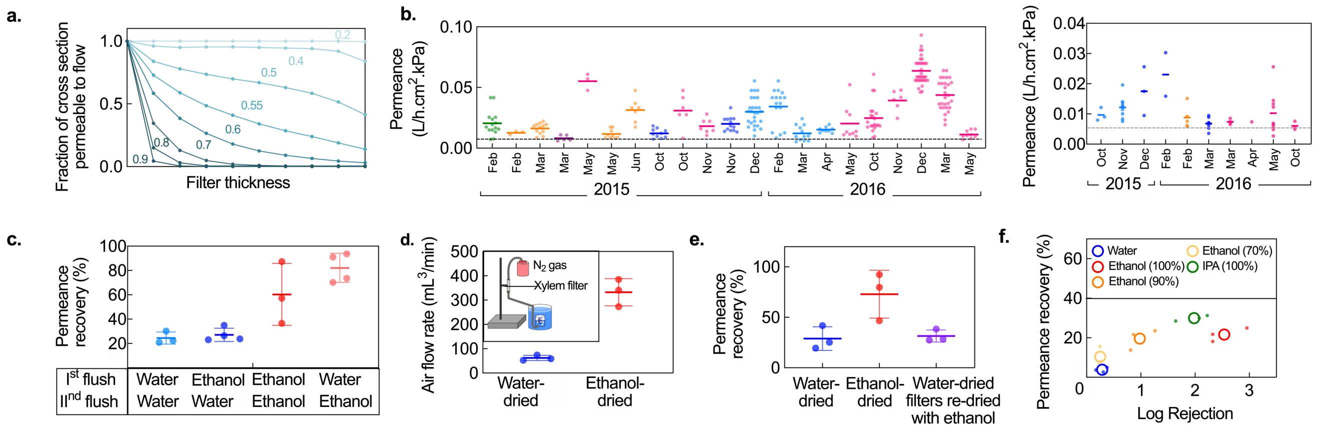

Supplementary Fig. 1| Mechanism of ethanol-based dry preservation (data reported with 1 cm diameter, 0.25-inch thick Eastern white pine filters, unless otherwise specified)**. a,** Variation of the filter cross-section that is permeable to flow (*i.e.*, connected to the top face) along filter thickness for different pit aspiration probabilities (probability values are color-coded and specified next to the corresponding curves). **b,** Permeance of fresh filters (n = 260 different filters) and ethanol-dried filters (n = 47 different filters) measured over a two-year period. Permeance of 95% of the filters exceeded 0.005 L/h.cm^2.kPa (shown by dotted line). Different colors denote different trees. Individual data points and their mean values are shown. Each data point represents a different filter (one measurement per filter). **c,** Presence of ethanol during drying is essential to recovery permeance on re-wetting, as seen from experiments where the filters were sequentially flushed with two fluids before drying. Individual data points and mean±s.d. are shown. n = 3, 4, 3, 4 different filters from left to right **d,** High permeance of ethanol-dried filters to air confirms that pit membranes pores are left open after ethanol treatment. Individual data points and mean± s.d. are shown. n = 3 different filters. **e,** Re-wetting water-dried filters with ethanol and then re-drying fails to improve permeance recovery (on wetting again with water). Individual data points and mean± s.d. are shown. n = 3 different filters. See Methods sub-section 'Rewetting of water-dried filters with ethanol' for experimental details. **f,** Effect of varying ethanol concentration and using isopropanol (IPA) on permeance recovery and rejection of xylem filters (filter thickness of 0.50 inches was used because the effect of alcohol treatment is more pronounced for thicker filters, facilitating comparison). Small, filled circles show individual data points while the large, open circles denote mean values. n = 3 different filters. Ethanol-water mixtures with concentration below 90% are ineffective in preserving permeance and rejection.

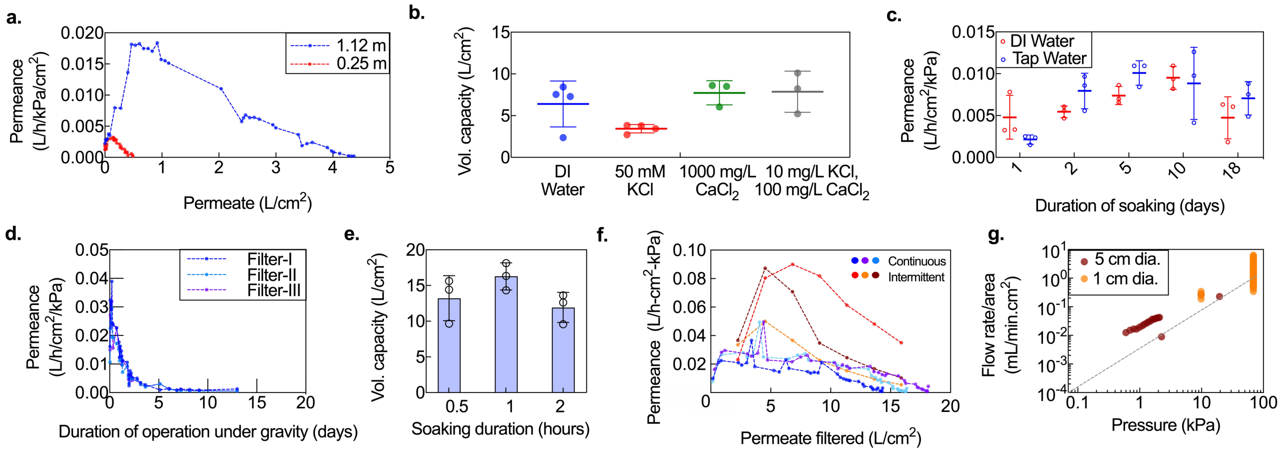

Supplementary Fig. 2| Mechanism of self-fouling and its mitigation (data reported with 1 cm diameter, 0.25-inch thick Eastern white pine filters, unless otherwise specified). **a,** Use of smaller gravitational head to drive flow fails to prevent permeance from declining to zero, suggesting that cavitation does not play a major role in filter blockage. Each data set corresponds to measurements with a single filter. **b,** Effect of addition of Ca^{2+} and K^{+} ions in DI water on volumetric (vol.) capacity. Mean±s.d.; *n* = 4, 4, 3, 3 different filters for DI water, 50 mM KCl, 1000 mg/L $CaCl_2$, and 10 mg/L KCl and 100 mg/L $CaCl_2$, respectively. Addition of ions to does not improve volumetric capacity. **c,** Extended soaking in DI or tap water does not result in blocking of filters. Mean±s.d., *n* = 3 independent measurements. **d,** Permeance decreases over time for DI water filtered under a 1 m gravitational head. **e,** Variation in volumetric (vol.) capacity with duration of soaking in hot water at 60-65°C. Mean±s.d., *n* = 3 different filters. **f,** Filters operated intermittently show a qualitatively similar trend as those operated continuously with values in the same range for both permeance and permeate filtered. Data for continuous operation were taken as is from Fig. 3f for filters treated with hot water soaking and ethanol drying. For intermittent operation, ginkgo filters (4 cm diameter, 0.375 inch thickness) were used (details in Methods sub-section 'Water flow rate and volumetric capacity measurements'). Different colors denote different filters. **g,** Flow rate normalized by area varies linearly with pressure for ethanol-dried filters with diameters 1 cm (*n* = 295 different filters; one measurement per filter) and 5 cm (*n* = 20 different filters; one measurement per filter). Dotted line shows a linear fit.

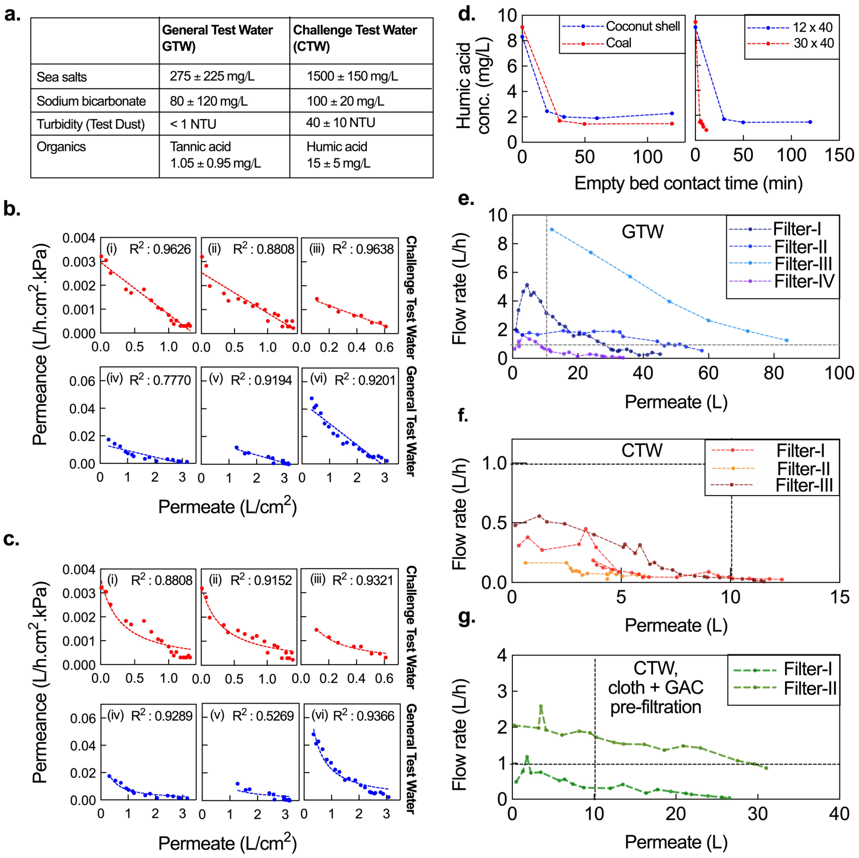

a.

	General Test Water (GTW)	Challenge Test Water (CTW)
Sea salts	275 ± 225 mg/L	1500 ± 150 mg/L
Sodium bicarbonate	80 ± 120 mg/L	100 ± 20 mg/L
Turbidity (Test Dust)	< 1 NTU	40 ± 10 NTU
Organics	Tannic acid 1.05 ± 0.95 mg/L	Humic acid 15 ± 5 mg/L

Supplementary Fig. 3| Fouling behavior of xylem filters. a, Composition of General Test Water (GTW) and Challenge Test Water (CTW). **b,c,** Complete blocking model (**b**) and cake filtration model (**c**) fits (dashed lines) to experimentally observed variation of permeance with volume filtered (symbols) for CTW (red) and GTW (blue). Each graph corresponds to measurements with a single filter. All measurement were performed under 1 m gravitational head with Eastern white pine filters (1 cm diameter, 0.375 inch thickness) except for plot (vi) where a ginkgo filter (4 cm diameter, 0.375 inch thickness) was used. **d,** Humic acid removal by coconut shell-based and coal-based GAC. **e,f,g** Variation of flow rate with volume filtered for 4 cm diameter, 0.375-inch thick ginkgo filters for GTW (**e**), CTW (**f**) and CTW with cloth and GAC pre-filtration (**g**). Different colors denote different filters.

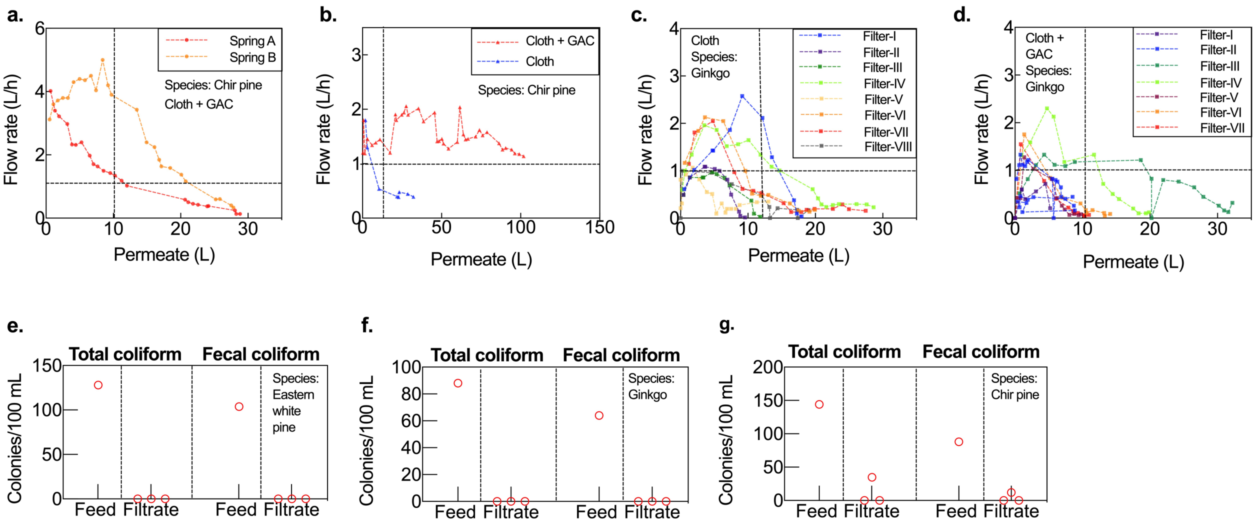

Supplementary Fig. 4| Field validation of xylem filters. a-d Variation of flow rate with capacity for (**a**) chir pine filters with water from two natural springs in Uttarakhand, with cloth + Granular Activated Carbon (GAC) pre-filtration (GAC design details in Supplementary Note 6), (**b**) chir pine filters with groundwater (obtained from tubewells) in Delhi with cloth and cloth + GAC pre-filtration, (**c**) ginkgo filters with municipal tap water in urban slums in Delhi with cloth pre-filtration, and (**d**) ginkgo filters with municipal tap water in urban slums in Delhi with cloth + GAC pre-filtration. Data is reported with 4 cm diameter, 0.375-inch thick filters processed using hot water soaking and ethanol treatment and operated under 1 m gravity head. **e-g** Removal of coliform bacteria by ethanol-preserved xylem filters (1 cm diameter, 0.25 inch thickness) operated under 1 m gravity head with water from a natural spring in Kith village, Uttarakhand. Filters were made from **e**) Eastern white pine, **f**) ginkgo and **g**) chir pine.

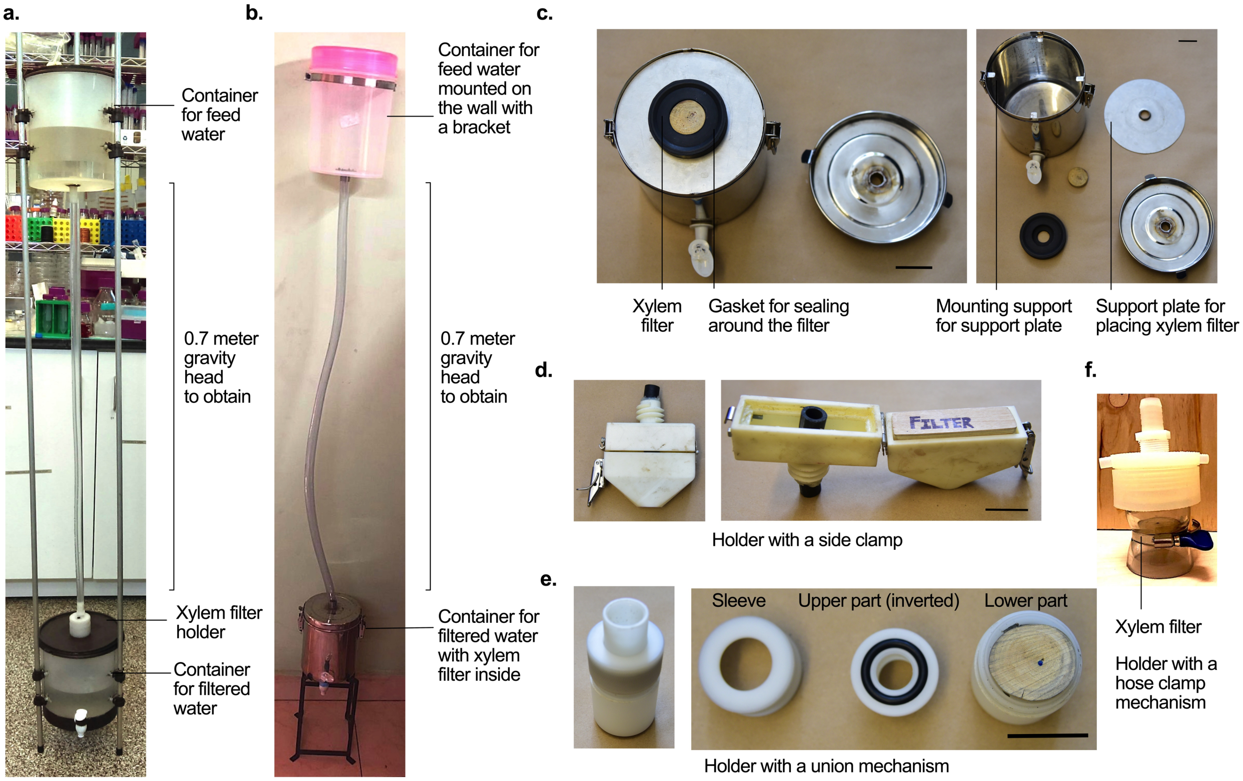

Supplementary Fig. 5| Xylem filter device prototypes a, Device prototype with which lab-based rejection tests and field-based capacity and flow rate tests were conducted. The filter holder is the same as the one shown in Fig. 6a, b and incorporates filters with 4 cm diameter and 0.375 inch thickness. **b,c,** Second version of a device prototype with a wall-mounted container for the feed water and a stainless steel receptacle for the filtered water. The xylem filter is inserted into a hole in a thick gasket, which seals the filter from the side. The gasket is clamped between a holder plate that rests on four mounting supports within the receptacle and the lid of the receptacle. Scale bar, 4 cm. **d-f,** Different filter holder mechanisms tested with users in addition to the screw-on mechanism shown in Fig. 6a,b. These include a holder with a latch on the side, a holder with a three-part union mechanism, and a holder with a hose clamp for sealing the filter from the side. See Supplementary Note 1 for details. Scale bar, 4 cm.

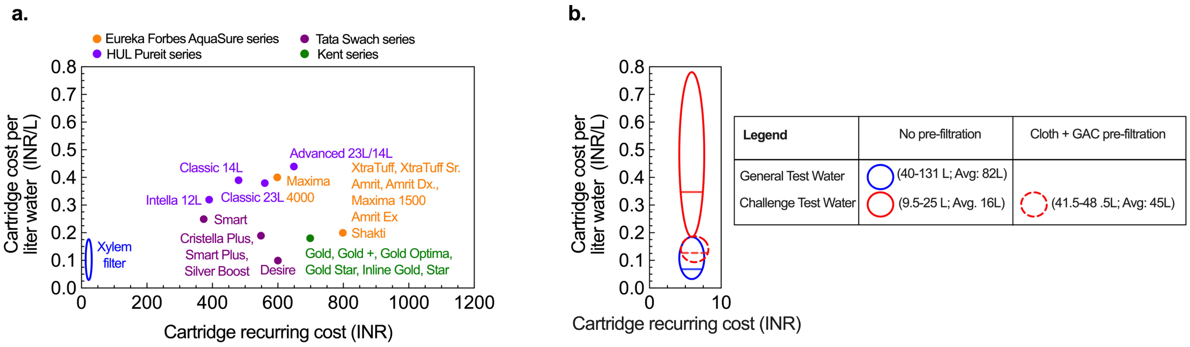

Supplementary Fig. 6| a, Cost comparison of xylem filters with commercial filters belonging to four major brands (denoted by different colors) in India shows the potential to offer unprecedentedly low cartridge replacement cost for a comparable or lower cost of cartridge per liter water filtered. Product names are included. INR stands for Indian Rupee. Capacity ratings for xylem filter are based on data obtained for ginkgo filters with World Health Organization (WHO)-prescribed General Test Water (GTW) (Fig. 4a, f); capacity for Hindustan Unilever (HUL) Pureit Series is rated at Total Dissolved Solids (TDS) of 300±10 ppm, pH of 7.5± 0.5, Total Organic Content (TOC) < 1 ppm, turbidity < 1 NTU. Capacity ratings for other filters are based on manufacturer-specific 'standard testing conditions'; in some cases, filter lifetime can be an order of magnitude lower if the water quality resembles WHO-prescribed Challenge Test Water (CTW). **b,** Xylem filter cartridge cost per liter water filtered for CTW is greater than that for GTW; use of cloth and Granular Activated Carbon (GAC) pre-filtration can reduce this cost substantially for a marginal increase in recurring cost. Capacity ranges and average (avg.) values corresponding to different water qualities and pre-filtration methods have been specified in the legend and are based on volumetric capacity measured for ginkgo filters in Fig. 4a, f (details of cost and capacity estimation have been provided in Supplementary Note 7). The horizontal lines indicate average estimated cartridge cost per liter (calculated as ratio of average filter price (INR 5.5) and average volumetric capacity specified in the legend).

Supplementary Notes

Supplementary Note 1: Criteria for practically useful filters

The criteria for practically useful water filters were formulated based on existing literature on design of point-of-use water treatment devices[1] and user research studies in the rural villages of Uttarakhand state and urban slums of New Delhi and Bengaluru in India conducted over a period of two years. In the user research studies, we engaged with over 1000 potential users, water filter manufacturers, and NGOs that distribute water filters through semi-structured/key informant interviews, focus group discussions, and design workshops to gather information on user preferences and response to xylem filters. The key findings from literature and user studies have been summarized below:

1. Although targets for microbiological performance of water filters and minimum flow rates for practical use of water filters have been well-documented in literature[2,3], filter lifetimes are relatively non-standardized and vary from 3-6 months for membrane-based filter cartridges to 1-2 years for ceramic filters[4–12]. Since xylem filters (in their present form) have a finite lifetime and require daily to weekly replacement that is very different from existing water treatment products, literature reports were not helpful in assessing user reception or setting filter lifetime targets for xylem filters. Consequently, we relied on data gathered from user studies (focus group discussions and individual household interviews) in India for developing a target filter lifetime. The preferred filter replacement frequency was found to be closely tied to other product attributes such as cost, ease of filter replacement, ease of availability of replacements, etc., but a minimum lifetime of one day was found to be critical for uptake. The filter capacity was thus determined based on a daily filter replacement frequency and estimates on the daily drinking water need for an average household. An average household in low-income countries of Asia and Africa comprises 4–5 members[13]. Given a recommended daily water intake for an individual of 2–3.5 L[14], the drinking water requirement for such households is 8-17 L per day. Xylem filters operated overnight (8–10 h) could meet this requirement if they have flow rates of at least 1–2 L/h; this flow rate is also the minimum useful flow rate reported in literature[3].
2. Household water treatment systems in resource-constrained areas should preferably be independent of electricity or tap pressure[1], which makes gravity-driven devices well-suited for such settings. The water head in these devices should be sufficient to achieve the desired flow rate, but not so high that it is inconvenient for users to fill the device with untreated water. 79 of the 100 participants in our user interviews reported that they would be comfortable with a total device height of 0.9–1 m.
3. Potential users in villages as well as slums mentioned that the natural appeal and the simplicity of xylem filters distinguished them from other products in the market. 40% of the 300 respondents cited these as the primary attributes they liked about xylem filter devices.
4. Consistent with literature[15,16], preliminary interactions with low-income households suggested that they prefer cheaper, frequent replacements to more expensive, long-term replacements. Of the 120 respondents in the urban slums of New Delhi, 50–60% reported an average household spending of $3 (INR 200) per week for purchasing reverse osmosis water cans and strongly indicated a willingness to switch to cheaper devices if they are available.
5. Users cited aesthetic appeal, ease of usage, and availability of devices at local shops as key factors for adoption of water filters.
6. Co-design workshops and interviews were conducted to identify holder designs that enable easy replacement of filter cartridges and are simple to use. Four different holder designs were tested: a) a holder for rectangular filters with an O-ring-based face-seal mechanism and side latch for clamping the filter in place (Supplementary Fig. 5d), b) a holder for circular filters with a union design comprising of three parts: a lower part for filter insertion, an upper part that connects to tubing and water reservoir from one end and face-seals against the filter using an O-ring insert on the other, and a sleeve that couples the upper and lower parts; this design allows for rotation of the lower part for inserting/removing the filter cartridge while maintaining the upper part and the associated tubing in a stationary position (Supplementary Fig. 5e) and prevents tube twisting, c) a holder for circular filters comprising of a plastic tubing for filter insertion and a hose clamp that seals against the sides of the filter; this mechanism enables utilization of the entire filter

surface area (Supplementary Fig. 5f), d) a two-part screw-on holder for circular filters where the filter is inserted into the lower part, and both parts containing O-rings inserts thread onto one another to face seal against the filter (Fig. 6b). Of the different holder mechanisms tested with 175 potential users, 64% of the respondents preferred the screw-on mechanism (design shown in Fig. 6b)) to other designs due to its relative ease of usage.

Supplementary Note 2: Simulation of percolation effects in xylem filters

A 2-D simulation model was constructed in MATLAB (Version R2017b 9.3.0) for 1 cm diameter, 1.5-inch thick xylem filters made from Eastern white pine. Assuming that, on average, the tracheids are 4-mm long and 40 μm in diameter[17,18], and that 80% of the filter cross section area is sapwood (typical for our filters), the model comprised a square lattice of 10×200 tracheids along the filter thickness and cross-section, respectively. For simplicity, we assumed that each tracheid was connected to four other tracheids (Fig. 2c) and that all tracheid-tracheid connections were either permeable (open) or aspirated (closed) with each tracheid-tracheid connection having an equal aspiration probability during drying. In general, the pit aspiration probability will depend on the condition of the neighboring tracheids and pit membranes, and may vary along the filter thickness depending on how the drying front propagates within the filter; such effects are not captured in the model.

This xylem structure was modeled using a 3-d matrix with the x and y indices representing the relative location of tracheids and the z indices representing the 'open' or 'closed' state (denoted by '1' and '0') of the four tracheid-tracheid connections after drying. A 10×100×4 matrix representing alternate tracheid columns accounted for all the tracheid-tracheid connections. To assign open/closed status to the tracheid-tracheid connections, uniform random numbers in the range of 0 to 1 were generated for each connection, and connections with numbers higher than the aspiration probability were designated to be open while the rest were designated closed. Depending on which tracheid-tracheid connections were open, tracheids that were connected to at least one tracheid in the top row of the filter through one or more open pathways were identified. To generate the graph in Fig. 2e, the fraction of such tracheids in the last row (row 10) was computed for different pit aspiration probabilities and averaged over 100 simulations. The insets in Fig. 2e were generated for one simulation at a given pit aspiration probability using the 'imagesc(xmap)' function in MATLAB. For Supplementary Fig. 1a, the fraction of tracheids in each row connected to the top row of the filter were computed and plotted against the row number on the x-axis (averaged over 100 simulations).

Supplementary Note 3: Ethanol treatment of xylem filters

Previous studies indicate that replacing water in xylem with non-aqueous liquids such as ethanol inhibits water-mediated adhesion between the pit membrane and cell wall, preventing pit aspiration during drying[19]. However, whether ethanol plays a role in deactivating any live processes critical for preventing adhesion is unknown. To investigate this possibility, ~1 cm diameter, 0.25-inch thick xylem filters made from Eastern white pine were flushed with ethanol and subsequently flushed with water and then dried. Their permeance (measured at 10 psi as described in Methods sub-section 'Water flow rate and volumetric capacity measurements') was compared to water-dried and ethanol-dried filters. The permeance recovery (ratio of permeance after and before drying) of these filters was comparable to 'water-dried' filters and significantly lower than 'ethanol-dried' filters (Supplementary Fig. 1c), demonstrating the absence of any residual effects of exposure to ethanol and discounting the possibility that live processes are involved in preventing pit aspiration.

SEM imaging and air flow rate measurements (see Methods sub-sections 'SEM imaging' and 'Air flow measurements') of ~1 cm diameter, 0.25-inch thick 'ethanol-dried' filters made from eastern white pine confirmed that the pit membranes remain suspended after ethanol treatment (Fig. 2g, Supplementary Fig. 1d), consistent with literature suggesting that ethanol prevents pit aspiration[19], as opposed to permitting pit aspiration with a weaker, reversible adhesion. The air permeance measurements were performed at low pressure (1 psi), where the mechanical force exerted on pit membranes is on the order of 1 μN, and given that 10 psi air pressure does not lead to recovery of permeance, it is insufficient to dislodge weakly adhered pit membranes from the cell wall. Furthermore, soaking 'water-dried' filters in ethanol failed to restore permeance (see Methods sub-section 'Rewetting of water-dried

filters with ethanol', Supplementary Fig. 1e), indicating that the adhesion between pit membrane and the cell wall was not reversed by exposure to ethanol.

Instead of ethanol, other alcohols like isopropanol can also be used for dry preservation, as they have similar effects on the drying mechanics of pit membranes (see Methods sub-section 'Treatment of xylem filters', Supplementary Fig. 1f). Ethanol-dried and isopropanol-dried eastern white pine filters (1 cm diameter, 0.50 inch thickness) showed similar permeance and rejection performance. To reduce costs, alcohols can be recycled and alcohol-water mixtures can be used. However, lowering alcohol concentration below 90% rendered the protocol ineffective (Supplementary Fig. 1f), presumably because the faster evaporation rate of alcohol relative to water results in an increase in the concentration of water as drying proceeds. The permeance and rejection performance of Eastern white pine filters (1 cm diameter, 0.50 inch thickness) treated with 70% ethanol was comparable to water-dried filters.

Supplementary Note 4: Alternate hypotheses for drop in permeance of xylem filters in the absence of contaminants

In addition to cellulosic material deposition, we considered the following hypotheses to explain the drop in permeance of xylem filters in the absence of external contaminants:

1. Filters could get blocked due to pit aspiration induced by the nucleation of gas bubbles (*i.e.*, cavitation) at the low-pressure end of the filter, where gas solubility is lower than the high-pressure end. However, operating ethanol-dried Eastern white pine filters (1 cm diameter, 0.25-inch thickness) at lower pressures to reduce the variation in solubility across the filter thickness failed to preserve permeance (Supplementary Fig. 2a), suggesting that the underlying mechanism is not cavitation-driven.
2. Previous studies have reported that the fibrils in the pit membranes swell to form a gel in the absence of Ca^{2+} and K^{+} ions normally present in sap[20,21], which could result in a loss of permeable membrane area leading to a drop in permeance. However, addition of Ca^{2+} and K^{+} salts to DI water at physiological concentrations found in plants failed to improve the capacity of ethanol-dried eastern white pine filters (1 cm diameter, 0.25 inch thickness), ruling out this hypothesis (Supplementary Fig. 2b).

Supplementary Note 5: Fouling model for xylem filters

We considered three different mechanisms that are conventionally used to model fouling in micro- and ultra-filtration membranes[22]:

<u>Complete blocking model</u>: In the complete blocking model, each foulant particle blocks a pore completely without depositing over previously deposited particles. The number of open pores, p, therefore decreases linearly with the filtrate volume, $\mathcal{V}$, as follows[22]:

$$p = p_0 - \rho_n \mathcal{V} \qquad \text{(Supplementary Equation 1)}$$

where, p_0 is the number of initially open pores in the filter, and ρ_n is the number density of foulant particles per unit volume of the feed. As the flux through the filters is directly proportional to the number of open pores, the expression above can be written as follows:

$$J = J_0\,(1 - K_{cb}\mathcal{V}) \qquad \text{(Supplementary Equation 2)}$$

where, J and J_0 represent the flux through filter in the fouled and initial state, respectively, and K_{cb} is called the blocking constant for the complete blocking model.

<u>Intermediate blocking model</u>: Here, the foulant particles deposit directly on the pores blocking them completely, or land on previously deposited foulant particles[22]. The number of open pores therefore decays exponentially with the filtered volume[23]:

$$p = p_0 e^{-K_{ib}\mathcal{V}} \quad \text{(Supplementary Equation 3)}$$

where, K_{ib} is the intermediate blocking constant. The flux can be written as follows:

$$J = J_0 e^{-K_{ib}\mathcal{V}} \quad \text{(Supplementary Equation 4)}$$

Cake filtration model: In cake filtration, the foulant particles deposit on the pores forming a permeable cake. The hydraulic resistance of this cake layer is in series with the resistance of the pores and increases linearly with the amount of foulant deposited (and hence with the filtered volume). The total resistance of each pore, R is then represented by the following equation:

$$R = R_{\text{pore}} + R_{\text{cake}} = R_{\text{pore}} + r\frac{\mathcal{V}}{p_0} = R_{\text{pore}}\left(1 + \frac{r}{R_{\text{pore}}}\frac{\mathcal{V}}{p_0}\right) = R_{\text{pore}}\left(1 + K_{cf}\mathcal{V}\right) \quad \text{(Supplementary Equation 5)}$$

where, R_{pore} is the resistance of the pores in the absence of fouling, R_{cake} is the resistance of the cake layer, and r is the resistance added by foulant deposition on the pore per unit volume of fluid filtered.
The flux, J, can then be written as follows:

$$J = \frac{\Delta P}{R} = \frac{\Delta P}{R_{\text{pore}}\left(1 + \frac{r}{R_{\text{pore}}}\frac{\mathcal{V}}{p_0}\right)} = \frac{J_0}{\left(1 + K_{cf}\mathcal{V}\right)} \quad \text{(Supplementary Equation 6)}$$

where, ΔP is the pressure difference applied across the filter.

Of the three models, the intermediate fouling model provided the best fit to experimental data for Challenge Test Water and General Test Water (Fig. 4d, Supplementary Fig. 3b,c). The models differed primarily near the filters' end-of-life, where the permeance gradually tailed-off in alignment with the intermediate blocking model whereas the complete blocking model predicted a much sharper, linear decline in permeance to zero, and the predictions of cake filtration model overshot experimentally observed values. These findings were further validated by SEM images of partially fouled *Ginkgo biloba* filters, where the pattern of foulant deposition was consistent with what would be expected in case of the intermediate fouling model (Fig. 4e).

Supplementary Note 6: Design of Granular Activated Carbon (GAC) column

GAC is a porous carbon-based material that removes contaminants (organic, as well as some inorganic compounds) by adsorption[24,25]. GAC is commonly used in municipal wastewater treatment plants as well as in household drinking water treatment[24,25]. The efficacy of contaminant removal depends on a) the carbon source, which determines the pore size and specific area and thus, the types of contaminant that can be removed, b) the size of GAC granules, which governs adsorption kinetics, and c) the contact time of feed water with GAC, which determines the time available for adsorption to take place[24].

To design an effective GAC pre-filtration column, we studied the adsorption characteristics of different commercially available GACs using humic acid as a model organic contaminant. Coal-based and coconut shell-based GACs with grain sizes of 0.6–2.4 mm, 0.4–1.7 mm, and 0.4–0.6 mm (characterized by sieving meshes of size 8×30, 12×40, and 30×40 respectively; 8×30 denotes granule sizes that pass through a mesh of size 8 but not through a mesh of size 30) were studied. GAC granules were packed in a 5 cm diameter, 15 cm long cylindrical pre-filtration column and humic acid removal from DI water at the same alkalinity and salinity as CTW was measured for different empty bed contact times (time for which the water is nominally in contact with the GAC; calculated by dividing the volume of the column by flow rate). Coal-based GAC was able to adsorb more humic acid for the same contact time than coconut shell-based GAC, and grains with a mesh size of 30×40 showed 20× faster adsorption than those with a mesh size of 12×40 (Supplementary Fig. 3d). Based on these results, we designed a coal-based

GAC column (5 cm diameter, 15 cm length) consisting of 30×40 granules that could be operated at flow rates of ~2 L/h (flow rates controlled by a valve) to reduce humic acid concentration in CTW to < 1 mg/L (details on cost and replacement frequency of GAC are provided in Supplementary Note 7).

Supplementary Note 7: Cost of manufacturing xylem filters
The manufacturing costs of xylem filters (5 cm diameter and 0.375-inch thickness) are divided into four categories: raw material, wood processing, packaging, and transportation.

1. Raw material: Globally, the typical price of commercial softwood ranges from 40-140 USD per cubic meter[26]. Assuming that a filter uses ~20 cm^3 of wood, the cost of wood required for fabricating a filter is 0.08-0.30¢. It is to be noted that the sapwood used for making the filters is a waste by-product of the timber industry due to its low mechanical strength and durability, and may be available at lower cost.

2. Processing: The processing of xylem filters involves cutting the wood into desired sizes, soaking the filter in hot water, soaking the filters in alcohol and drying the filters. Assuming that filters are fabricated in batches of 500, the cost estimates for each of these steps has been provided below:
a. Wood cutting cost: This comprises of three components:
- Equipment cost for cutting each filter calculated at a band saw cost of 900 USD[27,28], equipment lifetime of 5 years, duration of operation of 8 hours/day for 5 days a week, and a processing time of 10 s per filter: 0.03¢
- Utilities cost calculated for a band saw with a power rating of ~1 kW[28] at an electricity tariff of 10¢ per kWh[29]: 0.03¢
- Labor cost calculated at $5 per day (cost based on field visits in India; could vary with geography) and a processing time of 5 s per filter: 0.09¢
- Total wood cutting cost per filter: 0.15¢

b. Hot water soaking:
- Cost of water calculated based on a water requirement of 0.5 L for every filter (can be further optimized) and an average water tariff of 1.98 USD per cubic meter[30]: 0.1¢
- Cost of utilities for hot water treatment assuming a heat consumption of 100 kJ, gas stove energy efficiency of 10%, and charges of 8 ¢/h for a gas stove with a standard capacity of 10,000 kJ/h: 0.8¢
- Labor cost assuming a labor charge of $5 per day and time requirement of ~3 s for handling: 0.05¢
- Total cost of hot water treatment: 0.95¢

c. Alcohol treatment: While filters were manufactured primarily using ethanol in this study, other alcohols like methanol and isopropanol may also be used. Methanol is less expensive than ethanol. However, since methanol is toxic, appropriate food safety standards should be consulted to determine the grade of methanol that can be used, and the level of residual methanol in the dried filters should be maintained within the permissible limits for human consumption as prescribed by safety standards[31].
- Cost of methanol treatment calculated at as requirement of 0.1 L per filter at a price of $0.6-1/kg[32]: 5-8¢
- Labor charge for alcohol treatment estimated based on a labor charge of $5 per day and processing time of 3 s per filter: 0.05¢
- Cost of alcohol treatment for 1 filter: 5.05-8.05¢
- The cost for alcohol treatment can be substantially reduced if the alcohol is reused/recycled. Compared to immersion in a single alcohol bath, the alcohol consumption may be reduced by immersing the filters successively in multiple alcohol baths of smaller volumes.

Total processing cost: ~6-9¢

3. Packaging: Cost of packaging filters individually in heat-sealed HDPE (high density polythene) pouches are estimated as follows:
- Cost of plastic required for packaging calculated using HDPE price of $2-5/kg[33], HDPE density of 930-970 kg/m^3 [34], plastic requirement of ~1 cm^3 per filter (200 cm^2 area, 50 μm thickness): 0.19-0.5¢
- Cost of heat-sealing the plastic around the filter calculated based on a cost estimate of $40 for the heat

sealer[35], equipment lifetime of 1 year, operation duration of 8 h/day for 5 days a week and packaging time of 5 s per filter: 0.008¢
- Labor cost for filter packaging estimated based on a labor wage of $5 per day and processing time of 5 s per filter: 0.09¢

Total packaging cost: 0.3-0.6¢

<u>4. Transportation:</u> Assuming no infrastructure for transportation, the filters can be sent by post from the manufacturing location to the customer base. The postal charges for sending filters as a package of 200 is 0.03–0.07¢ (based on field visits in India, will vary with geography).

The total estimated cost of the xylem filters is therefore **6.5-10¢ (INR 4.5–7)**. As evidenced by the performance studies on the field, the capacity of each filter could range anywhere between 10–100 L, depending on the water quality and nature of pre-filtration.

If used, a cloth filter would add $1 to the cost (taken from report of surveys conducted in India[36]) and could be washed or replaced once it is dirty.

The GAC needs replacement after its adsorption capacity is expended, the frequency of which depends on the amount of GAC used, water quality, and adsorption capacity of the GAC. A GAC column for pre-filtration is expected to cost USD 0.67–5 per 1000 L of water filtered and require a replacement every 1.5-6 months based on the following estimates:

- Cost of coal-based GAC in a column measuring 5 cm in diameter, 15 cm length (as described in Supplementary Note 6) calculated using the following estimates:
  - (In the US) Price and density of USD 7-10.5 per kg and 0.5 g/cm^3 respectively for coal-based GAC[37]: USD 2-3
  - (In India) Price and density of INR 100 per kg and 0.5 g/cm^3 respectively for coal-based GAC[38]: INR 15
- Adsorption capacity of GAC for organic matter (estimated based on equilibrium adsorption data for dissolved organic matter (DOM) and humic acids on coal-based GAC (granule size 0.3-0.4 mm) reported in literature; input concentrations of DOM/humic acid were in the range of 5.5-12 mg/L; adsorption capacity of GAC was determined based on the amount of humic acid adsorbed per unit mass of GAC at saturation, i.e., when the residual concentration in the treated solution equaled initial concentration[39]): 30-100 mg (DOM or humic acid)/ g GAC
- Total amount of organic matter that can be adsorbed by a coal-based GAC column with aforementioned dimensions: 4.5-12 g for DOM/humic acid
- Volumetric capacity of GAC column (total volume of water the GAC column can process while effectively removing organic contaminants) estimated based on adsorption capacity at different DOM/humic acid concentrations in water ranging from 5.5 mg/L to 12 mg/L[39]: ~500 – 2000 L
- Cost of GAC column per 1000 liters of water: USD 0.5-3 or INR 0.008-0.03
- Replacement frequency of GAC columns assuming each household comprises of 4 members, each consuming 3 L of drinking water per day[13,14]: 1.5-6 months

In Supplementary Fig. 6, the recurring (cartridge) cost of a xylem filter is compared with different commercial filters in the Indian market. The rated capacity and the costs of filtration units and replacement cartridges was obtained from product websites[4–11]. The cartridge cost was normalized by the rated capacity to obtain the cost per liter of filtered water. The recurring cost for xylem filters was estimated using the above xylem filter cost (INR 4.5–7). Cost of cartridge per liter water filtered was obtained by dividing the recurring cost with the filter capacity for different water qualities. Capacity was estimated by using the data presented for ginkgo filters in Fig. 4a, f to obtain the permeate filtered per unit area for different water qualities and pre-filtration methods and then scaling the permeate per unit area for filters with 5 cm diameter; capacity ranges and average values for different scenarios

have been specified in the legend. The average cartridge cost per liter water filtered for each scenario was estimated by dividing the average filter price (INR 5.5) with the corresponding average volumetric capacity. The cost of the GAC column was evenly amortized across the cartridge replacement costs as follows:

- Assuming that the daily drinking water requirement for an average household is 8-17 L per day (as estimated in Supplementary Note 1), the replacement frequency of the xylem filter cartridge was estimated for different water qualities based on the corresponding filter capacity (as specified in the legend for Supplementary Fig. 6, based on data presented in Fig. 4a, f).
- The total number of xylem filter cartridge replacements required over the lifetime of the GAC column (1.5-6 months) was estimated.
- The cost of the GAC column was evenly amortized across the total number of replacements and added to the filter cartridge cost.

Supplementary Note 8: Resource requirement for fabricating xylem filters and filtration devices

**Task**	**Resources needed**
**Xylem filter cartridge**	
Extraction of branches	A pruner or sickle could be used for cutting branches. A chain saw might be used for cutting trunks.
Peeling of bark	The bark in the branches can be usually peeled by hand. Gloves should be used to avoid contact with resin. Band saws/hand saws might be needed for removing the bark from trunks.
Cutting the wood to desired sizes	Wood can be cut using a band saw, cold saw or hand saw. The surface roughness of the filter will vary with the equipment used. *Smooth surfaces are* ***required*** *to prevent leaks while using filters with a face seal holder* (as shown in Fig. 6a, b), but surface roughness might be a cause of lesser concern when a side sealing mechanism is used (Fig. 5c). To obtain a smooth surface, filters should be cut with a cold saw.
Hot water treatment	Clean (tap) water, vessels for soaking the filters in water, stove (gas/electric/induction) or just fuel wood for heating the water to 60 °C, and a thermometer for monitoring temperature.
Alcohol treatment	Certified, food-grade alcohol (methanol, ethanol, etc.) with >99% purity and alcohol-compatible vessels for soaking the filters. The level of residual alcohol in dried filters should be maintained within the permissible limits for human consumption as prescribed by food safety standards[31].
Drying	Filters could be dried at room/ambient temperatures of 25–40°C or using an oven.
**Filtration device** (The table below enlists the resource requirement for the filter designs depicted in Fig. 6a, b and Supplementary Fig. 5a. Although these device were fabricated in the US, they are amenable to local manufacture in India. The containers, O-rings, valves, tubing, dispenser and metal rods used in the device are commonly available items and can be sourced locally. The processes necessary for device fabrication (cutting, drilling, injection molding, etc.) are also well-established in the manufacturing industry. The device design could also be tuned as per the local availability of resources. For example, the filtration device shown in Supplementary Fig. 5b, c was fabricated in India. The steel and plastic containers used in the device were purchased from a local shop whereas the rubber gasket used for mounting the filter was fabricated using a custom-designed mold at a local medium-scale machine shop. The cost of the device (purchase price of containers, cost of mold fabrication, rubber cost) was INR 800 (USD 11), which could be reduced substantially when manufacturing at scale.)	
Storing unfiltered water	Food-grade container with appropriate capacity should be used. In Fig. 6a and Supplementary Fig. 5a, the container capacity is 5 L.

Providing gravitational head	The container with the unfiltered water has to be placed at a suitable height to provide the gravitational head to drive the water through the filter (lower head may be compensated for by larger filter area). While this could be achieved in different ways, a couple of mechanisms have been illustrated in Fig. 6a, where the container is placed on a sturdy stand, and in Supplementary Fig. 5a, where the container is mounted on the wall using a bracket.<br>Food-grade tubing is needed to connect the container at the top to the filter holder.
Holding the filter in the device	Potential users in India highly preferred a holder with a screw-on mechanism (shown in Fig. 6a, b). The particular holder, designed for 5 cm diameter, 0.375-inch thick filters, was machined from High Density Polypropylene, but the design is amenable to mass manufacture using injection molding. The CAD files of the holder will be made available to readers upon reasonable request to the corresponding authors. The key criteria that determine the successful functioning of the holder include:<br>a) An O-ring of appropriate hardness, such that it conforms to the wood surface to seal it effectively. For the holder in Fig. 6a, b, silicone O-rings with a shore hardness of 70 (procured from The Hope Group in Massachusetts, USA) were used.<br>b) Appropriate depth and width of the O-ring grooves is critical. Excessively deep grooves compromise the ability of the O-ring to conform to the wood surface, while shallow grooves may cause the O-ring to fall out, making user handling difficult. Further, O-rings that are smaller than the holder diameter reduce the effective cross-section area available for filtration (while accommodating a larger size range of filters), but larger O-rings increase the chance of leakage (when variations in filter shape cause the O-ring to sit on or beyond the filter edge). For the holder in Fig. 6b, the depth and width of the O-rings was 76% and 124% of their thickness respectively (conventional design values are 80% and 120%) and the O-ring diameter was 25% smaller than that of the nominal diameter of the filter (as shown in the inset in Fig. 6c). The inner diameter of the holder was 4.3 cm and the O-ring was designed to seal a filter with a diameter 4 cm or more.<br>c) Preventing entrapment of air bubbles. Air trapped in the tubes and connectors can disrupt water flow. Using tubes and connectors with sufficiently large diameters and avoiding narrow constrictions in the flow pathway can avoid trapping of air. In Fig. 6a, 0.25 inch diameter tubes and the connectors were used, and a vent consisting of a small hole plugged with an insert made of styrene butadiene rubber (SBR) procured from McMaster Carr (part number 9545K38) was also provided to release any air trapped within the holder.
Controlling water flow from the top container	A valve may be used between the top container and the filter, such that users can turn off the water supply when the device is not in use or while replacing the xylem filter cartridge.
Storing the filtered water	Food-grade container with appropriate capacity and a lid to prevent re-contamination should be used. The container should also have a dispenser to access the filtered water. This container should be placed at a height of at least 10-15 cm above the floor such that glasses, bottles, or other utensils can be placed below the dispenser.
Filter stand	The filter stand in Fig. 6a was fabricated using 0.25-inch thick aluminum angle rods (90°). However, stands could be fabricated in several other ways.

Supplementary Note 9: Behavior change interventions for HWT adoption

Through field visits (see Methods sub-section 'Field Studies' for further details on sampling, data collection and analysis methods), we have identified that the following factors affect behavior change and sustained adoption of HWT methods amongst low-income communities in India (these factors are in alignment with existing literature on HWT adoption in developing countries and low-income population[40–46]):

- Perceived quality of water source
- Perceived health risk of consuming contaminated drinking water
- Prior history of practicing HWT
- Cultural practices associated with water collection, storage, and consumption
- Affordability
- Accessibility to HWT products
- Convenience of use of HWT products
- Level of social support from peers
- Level of positive, ongoing contact with local healthcare workers
- Incentive structure for HWT adoption
- Extent of information dissemination about the effectiveness of HWT methods and their local accessibility/availability

Based on the aforementioned factors, which are broadly applicable to HWT methods, we have identified specific behavior change interventions that could facilitate the adoption of xylem filters:

- Community-level WASH education and awareness campaigns (would involve educating users on indicators of water quality, health effects of consuming contaminated drinking water, and testing local water quality with potential users to determine whether water is safe for consumption; could be conducted in collaboration with local NGOs or healthcare workers).
- School-level education and awareness programs (could use xylem filters as effective educational tools for designing science experiments and engineering projects that capture the interest of students and raise awareness of WASH issues at a very early age, which could be a powerful way to drive behavior change).
- Effective marketing strategies and promotional programs to facilitate uptake of xylem filters (demonstrations, free/discounted product trials, incentive programs such as providing cell phone minutes, cash, engagement of local community leaders for product endorsement, etc.).
- Ensuring availability of filter devices and cartridges in local shops to enable easy access.
- Appropriate pricing of xylem filters (involves gathering data on user willingness to pay, benchmarking against locally available HWT methods, and analyzing costs for filter production and distribution).
- Designing filtration device for maximal ease of usage and gathering user feedback to identify improvements.
- Regular monitoring and engagement with households to encourage sustained usage (visits could be conducted in partnership with local NGO staff and health care workers).

Supplementary Note 10: Improvement in rejection ability due to fouling

Deposition of foulants on the pit membranes over the course of filter operation is likely to further improve rejection. We observed that *Ginkgo biloba* filters rejected 94.01±3.31% of 100 nm particles, and that the deposition of merely 0.13 mg of foulant (humic acid) per cubic centimeter of the filter volume improved the rejection to 98.73±0.41%.

Supplementary Note 11: Design guide for selecting tree species for making xylem filters

Gymnosperm xylem exhibits great morphological variability across and within species, and even within the same tree. A description of how the filter performance (*i.e.*, permeance and rejection) may vary with the structural characteristics of the xylem is provided below. Supplementary Data 1 list these characteristics in detail[17,18,47–58] and

also provides information on geographic availability, pricing, and decay resistance for a wide range of gymnosperms.

1. Flow rate: In general, for a given xylem structure without significant pit aspiration, the flow rate is expected to be proportional to sapwood area and driving pressure, and inversely proportional to filter thickness. In the absence of fouling, the flow rate through a xylem filter depends on:
    a. *Fraction of xylem-containing sapwood present in the filter cross-section:* Flow rate increases with sapwood area. Inclusion of impermeable heartwood in filters (*e.g.*, when a filter is made from a branch cross-section) can reduce the sapwood area available for filtration. Sections of branches used for filtration tend to have less effective area for filtration due to the impermeable heartwood present in the center. Filters made exclusively from the sapwood in trunks have their entire area available for filtration but are limited in size by the width of the sapwood in the trunk. Some tree species, like ponderosa pine (*Pinus ponderosa*), ocote pine (*Pinus oocarpa*), Douglas fir (*Pseudotsuga menziesii*), pond pine (*Pinus serotina*), red pine (*Pinus resinosa*), spruce pine (*Pinus glabra*), Virginia pine (*Pinus virginiana*), and sitka/yellow/western/silver spruce (*Picea sitchensis*) have a wider sapwood and are well-suited for creating large area filters[59].
    b. *Tracheid and pit membrane properties:* Wider and longer tracheids can yield higher flow rates[52]. A high fraction of the tracheid wall area covered by pit membranes and higher pit membrane porosity increase fluidic conductivity. The tracheid conductivity for some tree species is provided in Supplementary Data 1. The tracheid length plays a key role in determining filter thickness. Filters should ideally comprise not more than 2-3 tracheids along their thickness to minimize flow resistance without compromising rejection ability. Tracheid lengths could vary significantly within a tree. For example, tracheids in trunks are typically longer than those in stems, and tracheid length also decreases with tree height. However, tracheid lengths are typically less than 5.6 mm[60].
2. Rejection: The rejection of the pit membranes depends on the following parameters:
    a. *Pore size of pit membranes:* Smaller pores have better rejection performance. The pit membrane pore size varies considerably across and within a plant. The pores in latewood (summerwood) are typically smaller than those in earlywood (spring wood)[61]. Intra-species variability is most prevalent amongst pines[61], where reported pore size measurements vary from 200-400 nm[17,18,61]. The pit membranes in ancient gymnosperms (belonging to genus *Cycas* and *Welwitschia*), *Ginkgo*, and cedars (which belong to genus *Thuja* in the cypress family), are very dense and are expected to be capable of excellent rejection[61].
    b. *Resin canals/ducts:* Resin canals/ducts are cylindrical intercellular spaces in the xylem oriented in the axial (longitudinal) direction[62]. Their inner surface is lined by epithelial cells, which secrete resins for defense against pests and pathogens[63]. If not filled with resin, these canals could act as leakage pathways in xylem filters and hence, attention should be paid to their presence. They could be several centimeters in length and are typically longer in the trunks than branches[64,65]. Resin canals are generally present in *Picea* (spruce), *Larix* (larch), *Pseudotsuga* (Douglas fir), and *Pinus* (pine); those in pines being numerous, large and evenly spaced while the ones in spruces, larches, and firs are few, small, and evenly spaced[66,67]. These canals are generally absent in *Abies* (fir), *Tsuga* (hemlock), *Pseudolarix* (golden larch), *Cedrus* (cedar), *Taxus* (yew, caution: yews are toxic), *Juniperus* (juniper), *Cupressus* (cypress) and *Ginkgo* (ginkgo) unless formed in response to external stimuli or stress[66,68,69].
3. Preservation of xylem filters: Filters made from tree species whose pit membranes are more resistant to cavitation may be less susceptible to drying-induced loss of performance during operation. Prior studies suggest that the resistance to cavitation depends on tracheid properties, the thickness or the rigidity of the torus, and ratio of the torus diameter to that of the cell wall aperture[51]. Junipers and cypress-pines tend to have high resistance to cavitation[51].

In addition to the aforementioned characteristics, the following notes could be useful for filter manufacture across species:

1. **Selection of branches:** Branches that do not have leaves undergo inactivation of the xylem through a process called compartmentalization[70] and have lower permeance. Junctions (where branches connect to one another) have a bent xylem vasculature and a lower hydraulic conductivity[71]. The wood from such branches and junctions should preferably not be used for manufacturing filters. Further, branches or trunks that show signs of decay (black marks, spots, etc.) should also be avoided.
2. **Standardization of filter size:** Xylem filters made from branches suffer from variability in shape and size, presenting challenges for designing compatible filter holders that can house these filters and enable their practical use. Coring out fixed-diameter circular discs from branches could enable standardization; but such a process can result in wastage of the xylem-containing sapwood present in the periphery while preserving the impermeable heartwood in the center, thereby reducing the effective area available for filtration. Standardized filters with a high fraction of sapwood area can be manufactured by cutting rectangular or circular sections from the peripheral sapwood in tree trunks. Certain species like Ponderosa pine, which have wider sapwood in the trunk cross-section, are more suited for the creation of such filters[59]. It is to be noted that the length of xylem conduits could vary across trunks and branches; as a result, the filter thickness may need to be adjusted to maintain rejection performance[72]. 0.375-inch thick filters made from branches of Eastern White Pine showed 98.8±1.2% rejection of 1 μm microspheres. However, 0.5-inch thick filters made from partly-dried trunks (procured after two weeks of felling) also showed comparable rejection (98.57±0.86%), whereas 0.75-inch thick filters showed a rejection of 99.21%±0.73%.
3. **Safety:** Selection of tree species for filter fabrication should be preceded by a thorough investigation of the potential toxic effects of its sap and methods to eliminate them (if any). Sap of some trees is consumed by humans[73,74], and sapwood occurs naturally in surface waters. Plants belonging to genus *Pinus* (pine), *Tsuga* (hemlock), and *Picea* (spruce) have been characterized as non-toxic[75,76], though the needles of *Pinus ponderosa* (ponderosa pine) and *Pinus contorta* (lodgepole pine) and can be toxic to cattle during gestation[77,78]. The oil extract of genus *Juniperus* (juniper) are commonly used in cosmetics[76]. Plants that are known to be non-toxic include Norfolk pine (Australia hemlocks). All parts of plants belonging to genus *Taxus* (yews) and *Cycas* (cycads) are known to be poisonous[79]. *Abies balsamia* (balsam fir) is known to be a skin irritant, and the seeds of *Ginkgo biloba* (ginkgo) are known to be poisonous[79]. **Confirming that the sapwood is nontoxic is essential for use of the filters for filtering drinking water for human consumption.** In addition, appropriate safety certifications and approvals may be procured before distributing the filters for human use. International NSF/ANSI standards are commonly employed to certify point-of-use drinking water filters. Although not required, the following certifications could be useful before marketing the product:
    a. NSF/ANSI 53: This standard is used to certify health-related microbiological contaminant reduction claims.
    b. NSF/ANSI 42: This standard is used to certify the chemical and material safety of filters. We note that drinking water standards typically do not have health-related constraints on natural organic matter in water (although there are constraints related to aesthetics, such as color).

In the future, it may be useful to develop certifications specifically for xylem filters.

Supplementary Note 12: HWT for emergency use

During disasters and emergency situations such as floods, disease outbreaks, contamination of public water supply network, etc., access to safe drinking water is a major cause of concern. Some common methods employed to provide safe drinking water to the affected population under such situations include distribution of bottled/packaged water procured from government agencies or commercial vendors, delivering water through water tankers, using neighboring water systems and using water treatment systems at point-of-entry or point-of-use[80]. Point-of-use, household water treatment (HWT) methods can be useful during the acute phase of an emergency when responders cannot reach the affected population and in the recovery phase when longer term solutions are still under development[81]. Effective use of HWT treatment methods, including water filtration, has been shown to effectively reduce the incidence of water-borne diseases during emergencies[81]. Examples of studies where filtration methods were evaluated during emergencies include the following: use of ceramic filters after the 2004 tsunami in Sri Lanka[82] and 2003 floods in Dominican Republic[83], distribution of ceramic/biosand after the 2010 earthquake in

Haiti[84,85], and use of membrane/ceramic filters during an emergency in Pakistan in 2007[86]. The key learnings from these studies, in conjunction with guidelines enlisted in the Sphere handbook (the primary reference tool for NGOs, UN agencies, and governments to respond to emergencies and disasters) that can help guide filter design and its implementation for emergency use include the following: a) the device should be able to meet minimum drinking water requirement for survival, which is 2.5-3 liters per person per day[87] and meet the minimum drinking water quality requirements during emergencies (< 10 CFU/100 mL, turbidity < 5 NTU)[87], b) the target price for emergency filters can be benchmarked against reported costs of filtration devices distributed during emergencies; during the 2003 floods in Dominican Republic, ceramic filters had an upfront cost of $15 (though they were distributed free of charge) with $4.50 recurring cost every 6 months for candle replacement[83], c) major factors affecting HWT usage rates amongst the affected population included quality of source water, prior experience with using HWT methods, need for training associated with device use, availability of replacement parts, level of programmatic support, ease of portability of device, and the living environment (usage rates varied between people living in permanent shelters and those moving between temporary shelters)[82–84,86,88]. Based on findings presented in the manuscript, xylem filters could be designed to meet the performance and cost targets specified above and due to their light weight, could be easy to transport and distribute. Other factors which could affect product adoption such as user training, filter lifetime, supply and distribution strategy, and user-perceived need for product would need to be further investigated.

The plastic bag design depicted in Fig. 7b is a conceptual sketch. Plastic containers have often been distributed for safe water storage and bucket chlorination during disasters and emergencies[89–92]. Several membrane filters designed for emergency use are available commercially[86,93,94], and some of these are membranes are housed in a plastic bag/container[86,94]. The robustness and longevity of the plastic bags would depend on the use case; filters could be designed to last for a single use or for a few days, weeks or months with provisions for cartridge replacement.

Supplementary Note 13: Potential avenues for engagement of micro-enterprises and local communities in xylem filter manufacture and distribution

The key raw materials required for xylem filter manufacture include gymnosperm sapwood and food-grade alcohol, which are available in several locations across the globe. In contrast to conventional membrane-based filter cartridges, the manufacturing process for filter fabrication is relatively simple and has not been patented for ease of dissemination of the technology. Accessibility to raw materials, simplicity of the manufacturing process, and the open source nature of the technology can facilitate its uptake by entrepreneurs and NGOs interested in taking it to the users and create an opportunity for business models where local communities can be engaged in filter manufacture and distribution. For example, communities could be involved to source the right kind of wood, or to process discarded branches, and bring them to a manufacturing facility, to distribute manufactured filters within rural/slum communities (the strategy of training local community members in retail and marketing for product sales and last-mile delivery is commonly employed by several fast moving consumer goods companies[95,96]), collect used filters to make charcoal, etc., and in some cases to fabricate filters in manufacturing facilities. The involvement of local communities in filter production and distribution could also help raise awareness about the safe importance of drinking water and encourage adoption of water treatment methods.

An example of an NGO that has implemented a micro-enterprise based model for water filters is Potters for Peace. Potters for Peace is a non-profit organization that has facilitated the dissemination of ceramic filters across the globe by training potters in several low-income communities to make these filters in their local facility in a sustainable manner[97]. The organization has established best practices for ceramic filter manufacture, offers training programs for potters and also provides ground assistance with factory set-up worldwide. Such a model could serve as an inspiration and an example template for xylem filter dissemination.

Supplementary Table 1: Microbiological performance data for xylem filters*

E. coli rejection							
Sample Point	Influent concentration (CFU/L)	Effluent concentration (CFU/L)			Log removal		
		Filter-I	Filter-II	Filter-III	Filter-I	Filter-II	Filter-III
Start	3.60×10^{9}	1.02×10^{6}	2.40×10^{5}	3.65×10^{5}	3.548	4.177	3.995
75% permeance	3.85×10^{9}	2.14×10^{6}	3.10×10^{5}	2.60×10^{5}	3.256	3.095	4.171
50% permeance	4.10×10^{9}	3.50×10^{5}	4.80×10^{5}	1.45×10^{6}	4.069	3.932	3.452
25% permeance	5.20×10^{9}	4.20×10^{5}	5.60×10^{5}	2.70×10^{5}	4.093	3.968	4.285
**MS-2 phage rejection**							
Sample Point	Influent concentration (PFU/L)	Effluent concentration (PFU/L)			Log removal		
		Filter-I	Filter-II	Filter-III	Filter-I	Filter-II	Filter-III
Start	4.25×10^{8}	2.52×10^{5}	3.78×10^{5}	1.87×10^{5}	3.227	3.051	3.357
75% permeance	5.30×10^{8}	1.88×10^{5}	4.56×10^{4}	2.45×10^{5}	3.451	4.066	3.336
50% permeance	6.22×10^{8}	2.40×10^{5}	2.60×10^{4}	3.86×10^{4}	3.414	4.379	4.208
25% permeance	3.52×10^{8}	3.65×10^{4}	1.96×10^{4}	4.56×10^{4}	3.985	4.255	3.888
**Rotavirus rejection**							
Sample Point	Influent concentration (PFU/L)	Effluent concentration (PFU/L)			Log removal		
		Filter-I	Filter-II	Filter-III	Filter-I	Filter-II	Filter-III
Start	1.03×10^{7}	1.00×10^{2}	1.50×10^{2}	1.70×10^{2}	5.011	4.835	4.781
75% permeance	1.03×10^{7}	2.50×10^{2}	3.20×10^{2}	3.40×10^{2}	4.613	4.506	4.48
50% permeance	1.02×10^{7}	5.00×10^{2}	6.70×10^{2}	7.50×10^{2}	4.308	4.181	4.132
25% permeance	1.02×10^{7}	7.50×10^{2}	8.50×10^{2}	9.20×10^{2}	4.132	4.078	4.044

*Xylem filters (4 cm diameter, 0.375-inch thickness) made from ginkgo were operated under a 1.2 meter gravity head with General Test Water containing WHO-prescribed concentrations of *E. coli* ($\geq10^{6}$ CFU/mL) and MS-2 phage ($\geq10^{5}$ PFU/mL)[98] and NSF-prescribed concentrations of rotavirus ($\geq10^{4}$ PFU/mL)[99]. *E. coli* and MS-2 phage were dosed simultaneously in the same test solution while rotavirus removal was tested separately. The bacterial and virus removal was tested at the start of filter operation and when permeance declined to 75%, 50%, and 25% of the initial value. After the first sampling point at the start of filter operation, dust was added to the test solution to accelerate clogging[100,101] (refer to Methods section M20 for further details on test procedure). Flow rates corresponding to different sampling points have been specified in the table below.

Sample Point	Flow rates (mL/min)					
	E. coli and MS-2 rejection experiment			Rotavirus rejection experiment		
	Filter-I	Filter-II	Filter-III	Filter-I	Filter-II	Filter-III
Start	24.50	25.00	26.00	25.00	26.00	26.00
75% permeance	18.40	18.80	19.50	18.80	19.50	19.50
50% permeance	12.30	12.50	13.00	12.50	13.00	13.00
25% permeance	6.10	6.30	6.50	6.30	6.50	6.50

Supplementary Table 2: Water quality parameters for field tests

Parameter	Water Source			Desirable Test Limit (BIS)	Permissible limit (BIS)	Standard test method
	Spring A (Kith village, Uttarakhand)*	Groundwater (Delhi)	Municipal tap water (Delhi)			
pH	8.03			6.5-8.5	No relaxation	IS 3025 (Part 11, 2002)
Total Dissolved Solids (TDS), mg/L	49.1			500	2000	IS 3025 (Part 16, 2006)
Turbidity (NTU)	5.02	<2		1	5	IS 3025 (Part 10, 2006)
Total alkalinity (as CaCO3), mg/L	50			200	600	IS 3025 (Part 23, 2003)
Total hardness (as CaCO3), mg/L	40			200	600	IS 3025 (Part 21, 2002)
Fluoride, mg/L	0.9			1	1.5	APHA 22nd Ed.-4500-F-D
Nitrate, mg/L	0.5			45	No relaxation	APHA 22nd Ed.-4500-NO3-B
Sulfate, mg/L	Not detected			200	400	APHA 22nd Ed.-4500-SO42-E
Residual free chlorine, mg/L	Not detected			0.2	1	APHA 22nd Ed.-4500-CI-G
Taste	Agreeable			Agreeable	Agreeable	APHA 22nd Ed.-2160-C
Color	Not detected			1	5	APHA 22nd Ed.-2120-C
Conductivity, uS/cm	81.3			-	-	APHA 22nd Ed.-2510-B
Chloride, mg/L	8.3			250	1000	APHA 22nd Ed.-4500-Cl-B
Iron (as Fe), mg/L	0.5			0.3	No relaxation	APHA 22nd Ed.-3500-Fe-B
COD		246.01	7.71			IS 3025 (Part 58)
Associated figures	Fig. 5f, Supplementary Fig. 4a	Fig. 5f, Supplementary Fig. 4b	Fig. 5g, h, Supplementary Fig. 4c, d			

*Spring A is depicted by a red circle in Fig. 5f. Water quality data for Spring B (Bhainswari village, Uttarakhand) denoted by the orange circle in Fig. 5f is unavailable. Data for Supplementary Fig. 4e-g was obtained using the water from Spring A (Kith, Uttarakhand) but water quality parameters other than fecal and total coliform were not tested when the experiments were conducted.

Supplementary Data 1. Geographic availability, structural and degradation characteristics, and pricing of gymnosperms : Data provided separately as Excel file.

Supplementary References

1. Peter-Varbanets, M., Zurbrügg, C., Swartz, C. & Pronk, W. Decentralized systems for potable water and the potential of membrane technology. *Water Res.* **43**, 245–265 (2009).
2. World Health Organization (WHO). *Evaluating household water treatment options: Health-based targets and microbiological performance specifications*. https://www.who.int/water_sanitation_health/publications/2011/evaluating_water_treatment.pdf (2011).
3. Potters for Peace. Best Practice Recommendations for Local Manufacturing of Ceramic Pot Filters for Household Water Treatment. *Group* 187 (2011).
4. Hindustan Unilever Ltd. Hindustan Unilever non-electric water purification products. https://www.pureitwater.com/IN/pureit-water-purifiers/type/non-electric.
5. Kent. Kent gravity water purifiers. https://www.kent.co.in/water-purifiers/gravity-uf/.
6. Eureka Forbes. AquaSure gravity water purifiers. https://www.eurekaforbes.com/water-purifiers/technology/non-electric-gravity.
7. Tata. Tata Swach non-electric water purifiers. https://tataswach.com/pages/non-electric-purifier.
8. Hindustan Unilever Ltd. Order PureIt GermKill Kit. https://www.pureitwater.com/IN/order-gkk.
9. Forbes Eureka. Accessories. https://www.eurekaforbes.com/accessories.
10. Swach, T. Spares. https://tataswach.com/pages/spares.
11. Amazon. Kent Gold Optima Spare Kit. https://www.amazon.in/Kent-Gold-Optima-Spare-Kit/dp/B00SMFPJG0.
12. van Halem, D., van der Laan, H., Heijman, S. G. J., van Dijk, J. C. & Amy, G. L. Assessing the sustainability of the silver-impregnated ceramic pot filter for low-cost household drinking water treatment. *Phys. Chem. Earth* **34**, 36–42 (2009).
13. United Nations. *Household size and composition around the world*. vol. 2 http://www.un.org/en/development/desa/population/publications/pdf/ageing/household_size_and_composition_around_the_world_2017_data_booklet.pdf (2017).
14. Gandy, J. Water intake: validity of population assessment and recommendations. *Eur. J. Nutr.* **54**, 11–16 (2015).
15. Mahajan, V. & Banga, K. *The 86 percent solution: How to succeed in the biggest market opportunity of the next 50 years*. (Pearson Education, 2005).
16. Banerjee, A. V & Duflo, E. The Economic Lives of the Poor. *J. Econ. Perspect.* **21**, 141–168 (2007).
17. Wilson, J. P. & Knoll, A. H. A physiologically explicit morphospace for tracheid-based water transport in modern and extinct seed plants. *Paleobiology* **36**, 335–355 (2010).
18. Bannan, M. W. Length tangential diameter and length/ width ratio of conifer tracheids. *Can. J. Bot.* **43**, 967–984 (1965).
19. Comstock, G. L. & Côté, W. a. Factors affecting permeability and pit aspiration in coniferous sapwood. *Wood Sci. Technol.* **2**, 279–291 (1968).
20. Zwieniecki, M. A. & Secchi, F. Getting variable xylem hydraulic resistance under control: Interplay of structure and function. *Tree Physiol.* **32**, 1431–1433 (2012).
21. Zwieniecki, M. A., Melcher, P. J. & Holbrook, N. M. Hydrogel control of xylem hydraulic resistance in plants. *Science (80-.).* **291**, 1059–1062 (2001).
22. Iritani, E. A Review on Modeling of Pore-Blocking Behaviors of Membranes During Pressurized Membrane Filtration. *Dry. Technol.* **31**, 146–162 (2013).
23. Hermia, J. Blocking Filtration. Application to Non-Newtonian Fluids. in *Mathematical Models and Design Methods in Solid-Liquid Separation* 83–89 (Springer, 2012). doi:10.1007/978-94-009-5091-7_5.
24. Çeçen, F. Activated Carbon. in *Kirk-Othmer Encyclopedia of Chemical Technology* (2014). doi:10.1002/0471238961.0103200902011105.a01.pub3.
25. Pollard, S. J. T., Fowler, G. D., Sollars, C. J. & Perry, R. Low-cost adsorbents for waste and wastewater treatment: a review. *Sci. Total Environ.* **116**, 31–52 (1992).
26. Oregon Small Woodlands Association. Today's market fot wood: a global, national and local view. https://knowyourforest.org/sites/default/files/documents/Gordon Culbertson Global Wood Supply and

Prices.pdf (2018).
27. IndiaMart. Band Saw Wood Cutting Machine. https://dir.indiamart.com/impcat/wood-band-saw.html.
28. Grizzly Industrial Inc. Grizzly G0513ANV - 17" 2 HP Bandsaw - 35th Anniversary Edition. https://www.grizzly.com/products/Grizzly-17-2-HP-Bandsaw-35th-Anniversary-Edition/G0513ANV.
29. Wikipedia. Electricity Pricing. https://en.wikipedia.org/wiki/Electricity_pricing.
30. Wikipedia. Water Tariff. https://en.wikipedia.org/wiki/Water_tariff.
31. International Council for Harmonisation. Guidance for Industry Q3C. *U.S. Dep. Heal. Hum. Serv. Food Drug Adm.* **9765**, 1–8 (2017).
32. Alibaba. Food Grade Methanol. https://www.alibaba.com/showroom/food-grade-methanol.html.
33. Alibaba. HDPE packing roll. https://www.alibaba.com/products/F0/hdpe_packing_roll/---------------------------G--12-100020959.html?spm=a2700.7724857.galleryFilter.57.bb38e75cx5vOCs.
34. Wikipedia. High-density polyethylene. https://en.wikipedia.org/wiki/High-density_polyethylene.
35. Alibaba. Plastic Heat Sealer. https://www.alibaba.com/showroom/heat-sealer.html.
36. Comprehensive Initiative on Technology Evaluation (Massachusetts Institute of Technology). Household Water Filter Evaluation, Suitability Report - Field Research in Ahmedabad, India. (2015).
37. Serv-A-Pure. Home > Resins & Media > Filter Media & Filter Gravel > Activated Carbons. https://www.servapure.com/Water-Washed-Bituminous-Coal-12-x-40_c_5709.html.
38. Indiamart. Granular Activated Carbon Coal-Based. https://dir.indiamart.com/search.mp?ss=granular+activated carbon&prdsrc=1.
39. Schreiber, B., Schmalz, V., Brinkmann, T. & Worch, E. The effect of water temperature on the adsorption equilibrium of dissolved organic matter and atrazine on granular activated carbon. *Environ. Sci. Technol.* **41**, 6448–6453 (2007).
40. Daniel, D., Marks, S. J., Pande, S. & Rietveld, L. Socio-environmental drivers of sustainable adoption of household water treatment in developing countries. *npj Clean Water* **1**, 1–6 (2018).
41. Hunter, P. R. Household water treatment in developing countries: Comparing different intervention types using meta-regression. *Environ. Sci. Technol.* **43**, 8991–8997 (2009).
42. Inauen, J., Hossain, M. M., Johnston, R. B. & Mosler, H. J. Acceptance and Use of Eight Arsenic-Safe Drinking Water Options in Bangladesh. *PLoS One* **8**, e53640 (2013).
43. Lantagne, D. S., Quick, R. & Mintz, E. D. Household water treatment and safe storage options in developing countries: a review of current implementation practices. *Navig* **99**, 17–38 (2006).
44. Loharikar, A. *et al.* Long-term impact of integration of household water treatment and hygiene promotion with antenatal services on maternal water treatment and hygiene practices in Malawi. *Am. J. Trop. Med. Hyg.* **88**, 267–274 (2013).
45. Ram, P. K. *et al.* Bringing safe water to remote populations: An evaluation of a portable point-of-use intervention in rural Madagascar. *Am. J. Public Health* **97**, 398–400 (2007).
46. World Health Organization (WHO). *Scaling Up Household Water Treatment Among Low-Income Populations*. http://www.who.int/household_water/research/household_water_treatment/en/index.html. (2009).
47. Bailey, I. W. & Tupper, W. W. Size Variation in Tracheary Cells: I. A Comparison between the Secondary Xylems of Vascular Cryptogams, Gymnosperms and Angiosperms. *Proc. Am. Acad. Arts Sci.* **54**, 149–204 (1918).
48. Langdon, L. M. Stem Anatomy of Dioon spinulosum. *Bot. Gaz.* **70**, 110–125 (2002).
49. Greguss, P. *Xylotomy of the living cycads, with a description of their leaves and epidermis.* (Akademiai Kiado, 1968).
50. Chrysler, M. A. Vascular Tissues of Microcycas Calocoma. *Bot. Gaz.* **82**, 233–252 (1926).
51. Bouche, P. S. *et al.* A broad survey of hydraulic and mechanical safety in the xylem of conifers. *J. Exp. Bot.* **65**, 4419–4431 (2014).
52. Pittermann, J., Sperry, J. S., Hacke, U. G., Wheeler, J. K. & Sikkema, E. H. Inter-tracheid pitting and the hydraulic efficiency of conifer wood: The role of tracheid allometry and cavitation protection. *Am. J. Bot.* **93**, 1265–1273 (2006).
53. Delzon, S., Douthe, C., Sala, A. & Cochard, H. Mechanism of water-stress induced cavitation in conifers:

Bordered pit structure and function support the hypothesis of seal capillary-seeding. *Plant, Cell Environ.* **13**, 2101–2111 (2010).
54. Jansen, S. *et al.* Plasmodesmatal pores in the torus of bordered pit membranes affect cavitation resistance of conifer xylem. *Plant, Cell Environ.* **35**, 1109–1120 (2012).
55. Pittermann, J. & Sperry, J. Tracheid diameter is the key trait determining the extent of freezing-induced embolism in conifers. *Tree Physiol.* **23**, 907–914 (2003).
56. Terrazas, T. Origin and Activity of Successive Cambia in Cycas (Cycadales). *Am. J. Bot.* **78**, 1335–1344 (1991).
57. Ryberg, P. E., Taylor, E. L. & Taylor, T. N. Secondary phloem anatomy of Cycadeoidea (Bennettitales). *Am. J. Bot.* **94**, 791–798 (2007).
58. Scott, D. H. On the primary structure of certain palæozoic stems with the dadoxylon type of wood. *Trans. R. Soc. Edinburgh* **40**, 331–365 (1905).
59. Ross, R. J. & and Others. Wood handbook : wood as an engineering material. *USDA For. Serv. For. Prod. Lab.* **190**, (2010).
60. Hacke, U. G., Sperry, J. S. & Pittermann, J. Analysis of circular bordered pit function II. Gymnosperm tracheids with torus-margo pit membranes. *Am. J. Bot.* **91**, 386–400 (2004).
61. Bauch, J., Schultze, R. & Liese, W. Morphological variability of bordered pit membranes in gymnosperms. *Wood Sci. Technol.* **6**, 165–184 (1972).
62. Baas, P. *et al.* IAWA List of microscopic features for softwood identification. *IAWA J.* **25**, 1–70 (2004).
63. Wikipedia. Resin canal. https://en.wikipedia.org/wiki/Resin_canal.
64. Bannan, M. W. Vertical Resin Ducts in the Secondary Wood of the Abietineae. *New Phytol.* **35**, 11–46 (1936).
65. Reid, R. W. & Watson, J. A. Sizes, distributions, and numbers of vertical resin ducts in lodgepole pine. *Can. J. Bot.* **44**, 519–525 (1966).
66. Wu, H. & Hu, Z. H. Comparative anatomy of resin ducts of the Pinaceae. *Trees - Struct. Funct.* **11**, 135–143 (1997).
67. Conners, T. The first separation of softwood species. *Agric. Nat. Resour. Publ.* **107**, (2015).
68. Fahn, A., Zamski, E. The influence of pressure, wind, wounding and growth substances on the rate of resin duct formation in Pinus halepensis wood. *Isr. J. Bot.* **19**, 429–46 (1970).
69. Govina, J. K. Resin and resin canals in families and clones of Pinus radiata (D. Don). (University of Canterbury, Christchurch, New Zealand, 2017).
70. Rioux, D. Compartmentalization in Trees: New Findings During the Study of Dutch Elm Disease. in *Histology, ultrastructure and molecular cytology of plant-microorganism interactions* 211–225 (1996). doi:10.1007/978-94-009-0189-6_12.
71. Schulte, P. J. Branch junctions and the flow of water through xylem in Douglas-fir and ponderosa pine stems. *J. Exp. Bot.* **54**, 1597–1605 (2003).
72. Sarén, M. P. *et al.* Structural variation of tracheids in norway spruce (Picea abies [L.] Karst.). *J. Struct. Biol.* **136**, 101–109 (2001).
73. Kallio, H., Karpinnen, T. & Holmbom, B. Concentration of Birch Sap by Reverse Osmosis. *J. Food Sci.* **50**, 1330–1332 (1985).
74. Holman, M. B. & Egan, K. C. Processing Maple Sap with Prehistoric Techniques. *J. Ethnobiol.* **5**, 61–75 (1985).
75. Illinois Poison Center (IPC). Plant list - poison and non-poison. https://www.illinoispoisoncenter.org/Plant-List.
76. Johnson, W. Final report on the safety assessment of juniperus communis extract, juniperus oxycedrus extract, juniperus oxycedrus tar, juniperus phoenicea extract, and juniperus virginiana extract. *Int. J. Toxicol.* **20**, 41–56 (2001).
77. Agriculture Research Service (US Department of Agriculture). Poisonous plant research: Logan, UT. https://www.ars.usda.gov/pacific-west-area/logan-ut/poisonous-plant-research/.
78. Lee, S. T. *et al.* Development of enzyme-linked immunosorbent assays for isocupressic acid and serum metabolites of isocupressic acid. *J. Agric. Food Chem.* **51**, 3228–3233 (2003).

79. Nelson, L. S., Shih, R. D. & Balick, M. J. *Handbook of poisonous and injurious plants*. (New York Botanical Garden, 2007).
80. Bross, L. *et al.* Insecure security: Emergency water supply and minimum standards in Countries with a high supply reliability. *Water* **11**, 732 (2019).
81. Lantagne, D. & Clasen, T. Point-of-use water treatment in emergency response. *Waterlines* **31**, 30–52 (2012).
82. Casanova, L. M., Walters, A., Naghawatte, A. & Sobsey, M. D. A post-implementation evaluation of ceramic water filters distributed to tsunami-affected communities in Sri Lanka. *J. Water Health* **10**, 209–220 (2012).
83. Clasen, T. Household-Based Ceramic Water Filters for the Treatment of Drinking Water in Disaster Response: An Assessment of a Pilot Programme in the Dominican Republic. *Water Pract. Technol.* **1**, (2006).
84. Lantagne, D. S. & Clasen, T. F. Use of household water treatment and safe storage methods in acute emergency response: Case study results from Nepal, Indonesia, Kenya, and Haiti. *Environ. Sci. Technol.* **46**, 11352–11360 (2012).
85. Lantagne, D. & Clasen, T. Effective use of household water treatment and safe storage in response to the 2010 haiti earthquake. *Am. J. Trop. Med. Hyg.* **89**, 426–433 (2013).
86. Ensink, J. H. J., Bastable, A. & Cairncross, S. Assessment of a membrane drinking water filter in an emergency setting. *J. Water Health* **13**, 362–370 (2015).
87. Sphere Association and others. *The Sphere Handbook: Humanitarian Charter and Minimum Standards in Humanitarian Response*. vol. 1 (Practical Action, 2018).
88. Staveley, L. & Lantagne, D. *Oxfam Household Water Treatment Technical Brief.* (2008).
89. Sikder, M. *et al.* Effectiveness of water chlorination programs along the emergency-transition-post-emergency continuum: Evaluations of bucket, in-line, and piped water chlorination programs in Cox's Bazar. *Water Res.* **178**, 115854 (2020).
90. Ferron, S. *Enabling Access to Non-Food Items in an Emergency Resposne: A review of Oxfam programmes*. www.oxfam.org (2017) doi:10.21201/2017.0292.
91. Ali, S. I., Ali, S. S. & Fesselet, J. F. Evidence-based chlorination targets for household water safety in humanitarian settings: Recommendations from a multi-site study in refugee camps in South Sudan, Jordan, and Rwanda. *Water Res.* **189**, 116642 (2021).
92. Center for Disease Control and Prevention (CDC). The Oxfam Bucket. https://www.cdc.gov/safewater/oxfam-bucket.html#:~:text=The Oxfam Bucket (Oxfam),for use in program implementation.
93. GrifAid. GrifAid Water Filters. https://www.grifaid.org/our-filters.
94. Lifesaver. Lifesaver Cube. https://iconlifesaver.com/product/lifesaver-cube/?v=7516fd43adaa.
95. Neuwirth, B. Marketing Channel Strategies in Rural Emerging Markets: Unlocking Business Potential. *Online. Accessed July 21 2012.* 1–40 (2012).
96. Dolan, C., Johnstone-Louis, M. & Scott, L. Shampoo, saris and SIM cards: Seeking entrepreneurial futures at the bottom of the pyramid. *Gend. Dev.* **20**, 33–47 (2012).
97. Potters for Peace. Ceramic Water Filter Project. https://www.pottersforpeace.org/ceramic-water-filter-project.
98. World Health Organization (WHO). WHO Intetnational Scheme to Evaluate Household Water Treatment Technologies Harmonized Testing Protocol: Technology Non-specific. (2014).
99. NSF International. *NSF Protocol P231 - Microbiological Water Purifiers*. (2014).
100. NSF International. *NSF/ANSI 53: Drinking Water Treatment Units - Health Effects*. vol. 29 11–12 (2019).
101. World Health Organization (WHO). WHO International Scheme to Evaluate Household Water Treatment Technologies Harmonized Testing Protocol: Technology Non-Specific. 1–5 (2014).
