# Supplementary material for: Engineering and characterization of gymnosperm sapwood toward enabling the design of water filtration devices
Source: Nat Commun. 2021 Mar 25;12:1871. doi: 10.1038/s41467-021-22055-w (PMC7994624; doi:10.1038/s41467-021-22055-w)
Supplement: Supplementary file 3 — Description of Additional Supplementary Files [file 41467_2021_22055_MOESM3_ESM.pdf]

Description of Additional Supplementary Files

File Name: Supplementary Data 1
Description: This dataset contains information on geographic availability, structural and degradation characteristics, and pricing of gymnosperms.
