# Supplementary material for: Engineering and characterization of gymnosperm sapwood toward enabling the design of water filtration devices
Source: Nat Commun. 2021 Mar 25;12:1871. doi: 10.1038/s41467-021-22055-w (PMC7994624; doi:10.1038/s41467-021-22055-w)
Supplement: Supplementary file 5 — Reporting Summary [file 41467_2021_22055_MOESM5_ESM.pdf]

nature research

Corresponding author(s): Rohit Karnik, Krithika Ramchander

Last updated by author(s): 01/15/2021

Reporting Summary

Nature Research wishes to improve the reproducibility of the work that we publish. This form provides structure for consistency and transparency in reporting. For further information on Nature Research policies, see Authors & Referees and the Editorial Policy Checklist.

Statistics

For all statistical analyses, confirm that the following items are present in the figure legend, table legend, main text, or Methods section.

n/a	Confirmed	
☐	☒	The exact sample size (*n*) for each experimental group/condition, given as a discrete number and unit of measurement
☐	☒	A statement on whether measurements were taken from distinct samples or whether the same sample was measured repeatedly
☒	☐	The statistical test(s) used AND whether they are one- or two-sided<br>*Only common tests should be described solely by name; describe more complex techniques in the Methods section.*
☒	☐	A description of all covariates tested
☒	☐	A description of any assumptions or corrections, such as tests of normality and adjustment for multiple comparisons
☒	☐	A full description of the statistical parameters including central tendency (e.g. means) or other basic estimates (e.g. regression coefficient) AND variation (e.g. standard deviation) or associated estimates of uncertainty (e.g. confidence intervals)
☒	☐	For null hypothesis testing, the test statistic (e.g. *F*, *t*, *r*) with confidence intervals, effect sizes, degrees of freedom and *P* value noted<br>*Give P values as exact values whenever suitable.*
☒	☐	For Bayesian analysis, information on the choice of priors and Markov chain Monte Carlo settings
☒	☐	For hierarchical and complex designs, identification of the appropriate level for tests and full reporting of outcomes
☒	☐	Estimates of effect sizes (e.g. Cohen's *d*, Pearson's *r*), indicating how they were calculated

Our web collection on statistics for biologists contains articles on many of the points above.

Software and code

Policy information about availability of computer code

Data collection	No code was used for data collection.
Data analysis	A Matlab (Version R2017b 9.3.0) code was developed to evaluate the dependence of permeance on xylem filter thickness for dried filters. The simulation setup and the parameters used have been provided in the Methods section and the code will be made available to readers upon reasonable request to the authors. The graphs in main and supplementary figures were generated using GraphPad Prism (Version 8.4.3) and assembled on Microsoft Power Point (Version 16.37). The mean values, standard deviations and fits were generated using in-built functions in the software. The p values for Figures 2f and 2h were generated using Microsoft Excel (Version 16.37).

For manuscripts utilizing custom algorithms or software that are central to the research but not yet described in published literature, software must be made available to editors/reviewers. We strongly encourage code deposition in a community repository (e.g. GitHub). See the Nature Research guidelines for submitting code & software for further information.

Data

Policy information about availability of data

All manuscripts must include a data availability statement. This statement should provide the following information, where applicable:

- Accession codes, unique identifiers, or web links for publicly available datasets
- A list of figures that have associated raw data
- A description of any restrictions on data availability

All data that support the findings of this study are available in the main text, figures, and supplementary information. Field study results are presented in aggregate to protect privacy of survey respondents. Data for number of diarrheal deaths shown in Fig. 1d was obtained from the Global Health Observatory data repository managed by the World Health Organization (URL (accessed on 14th June 2019): https://apps.who.int/gho/data/view.main.INADEQUATEWATERv?lang=en, Dataset title: 'Number of diarrhea deaths from inadequate water (2016)' within the section 'Burden of disease from inadequate water in low- and middle-income countries'.

Field-specific reporting

Please select the one below that is the best fit for your research. If you are not sure, read the appropriate sections before making your selection.

☐ Life sciences ☒ Behavioural & social sciences ☐ Ecological, evolutionary & environmental sciences

For a reference copy of the document with all sections, see nature.com/documents/nr-reporting-summary-flat.pdf

Behavioural & social sciences study design

All studies must disclose on these points even when the disclosure is negative.

Study description	The overall goal of the study was to understand user preferences with regards to household water treatment to inform xylem filter design and gauge user response towards xylem filters.<br><br>Specifically, the study focused on gathering information on the following topics:<br><br>1. Common water sources used by low-income communities in India<br>2. User perception of water quality and its key determinants<br>3. User experience with household water treatment (HWT) methods/products<br>4. User perceived need for HWT technologies and barriers for their adoption<br>5. User desired attributes in a HWT device and a xylem-based filtration device, and willingness to pay<br>6. User preference for filter holder design<br><br>The data collected from human subjects were generally qualitative, but some of the data were quantitative. For the qualitative data, qualitative content analysis was performed on the data to identify themes and sub-themes. For the quantitative data, descriptive statistics were generated.
Research sample	The research sample includes low income rural households in the mountainous state of Uttarakhand, India and the urban slum households from Bangalore and Delhi and other stakeholders in the water filter supply chain, including, filter vendors, manufacturers, NGO staff, and local health officials.
Sampling strategy	Different sampling strategies were followed for different data collection methods. In general, we followed the guideline of 20-30 subjects per segment to identify needs through interviews (Griffin, A.J. and Hauser, J.R. (1993) The voice of the customer. Marketing Science, 12(1)). Potential segments with potentially different needs were stratified based on a variety of factors including the geography, types of water sources, proximity to town, and heterogeneity of the population in terms of water related practices. Based on these criteria, locally based partner organizations helped us select a set of villages. Within those villages, for semi structured interviews, simple random sampling was used. For focus group discussions and design workshops, a combination of non-probability sampling techniques such as convenience sampling and snowball sampling techniques were used. For key informant interviews, the team used a purposeful sampling strategy to select participants.<br>Data saturation was also considered in the studies. With the help of local experts, we assessed the homogeneity of the population with regards to water usage practices and at a point when no new information or themes were observed in the data, it was considered data saturation.
Data collection	A few different methods were used to collect data including semi structured interviews, key informant interviews, focus group discussions, and design workshops. Pen and paper were used to record the data. Photos were taken and voice recorders were also used with the participant consent. A translator was present during data collection. Researchers were not blind to experimental condition and study hypothesis.
Timing	Cohort 1: Jan 9- Jan 28, 2017, Cohort 2: Aug 6- Aug 23, 2017, Cohort 3: Jan 6- Jan 14, 2018, Cohort 4: May 21- June 5, 2018, Cohort 5: Sept27- Oct 9, 2018.
Data exclusions	No data was excluded from the studies.
Non-participation	One participant in the data collection cohort 4 declined participation. The reason was that the participant was a female from a conservative Muslim family and she did not feel comfortable to answer the questions in the presence of a male researcher.
Randomization	Participants were not allocated into experimental groups.

Reporting for specific materials, systems and methods

We require information from authors about some types of materials, experimental systems and methods used in many studies. Here, indicate whether each material, system or method listed is relevant to your study. If you are not sure if a list item applies to your research, read the appropriate section before selecting a response.

Materials & experimental systems

n/a	Involved in the study
☒	☐ Antibodies
☒	☐ Eukaryotic cell lines
☒	☐ Palaeontology
☒	☐ Animals and other organisms
☐	☒ Human research participants
☒	☐ Clinical data

Methods

n/a	Involved in the study
☒	☐ ChIP-seq
☒	☐ Flow cytometry
☒	☐ MRI-based neuroimaging

Human research participants

Policy information about studies involving human research participants

Population characteristics	See above.
Recruitment	Sampling instructions were given to the local research partners and they recruited the participants as majority of the participants were the beneficiaries of those partner organizations. Recruitment happened through phone call and/or meeting. While the partner organizations followed our sampling strategy, there still may have been some bias in segment and/or participant selection.
Ethics oversight	Committee on the Use of Humans as Experimental Subjects (COUHES) at Massachusetts Institute of Technology (MIT) is the IRB that reviewed and approved the user research study protocol. Protocol number is 1612798762.

Note that full information on the approval of the study protocol must also be provided in the manuscript.
